# Publisher Correction to: Military Medical Research, volume 6

**DOI:** 10.1186/s40779-019-0215-8

**Published:** 2019-08-07

**Authors:** Military Medical Research

**Affiliations:** London, UK

**Publisher correction to: Military Medical Research, volume 6**

An error occurred during the publication of a number of articles in *Military Medical Research*. Several articles were published in volume 6 with a duplicate citation number.

In this correction article the old and new citation metadata are published in Table 1.

**Table 1:** Overview of incorrect and correct citation metadata

DOI	Incorrect citation number	Correct citation number
10.1186/s40779-019-0206-9	1	19
10.1186/s40779-019-0208-7	2	20
10.1186/s40779-019-0209-6	4	18
10.1186/s40779-019-0210-0	3	17

The original articles have been updated. The publisher apologizes for the inconvenience caused to our authors and readers.

